# Supplementary material for: Power supply disruptions deter electric vehicle adoption in cities in China
Source: Nat Commun. 2024 Jul 18;15:6041. doi: 10.1038/s41467-024-50447-1 (PMC11255310; doi:10.1038/s41467-024-50447-1)
Supplement: Supplementary file 1 — Supplementary Information [file 41467_2024_50447_MOESM1_ESM.pdf]

Supplementary Information

Power supply disruptions deter electric vehicle adoption in cities in China

Yueming (Lucy) Qiu [*, #, 1], Nana Deng[*,2,3], Bo Wang[#,3,4], Xingchi Shen [5] ,

Zhaohua Wang[#,2,3], Nathan Hultman [1], Han Shi[3,4], Jie Liu[6], Yi David Wang[7]

[1] School of Public Policy, University of Maryland at College Park, College Park, MD, United States, 20742

[2] School of Economics, Beijing Institute of Technology, Beijing, China, 100081

[3] Digital Economy and Policy Intelligentization Key Laboratory of Ministry of Industry and Information Technology, Beijing, China, 100081

[4] School of Management, Beijing Institute of Technology, Beijing, China, 100081

[5] Yale School of the Environment, Yale University, New Haven, CT, United States, 06511

[6] Institute of Technology for Carbon Neutrality, Shenzhen Institute of Advanced Technology, Chinese Academy of Sciences, Shenzhen, China, 518000

[7] Department of economics, Virginia Tech, Blacksburg, VA, United States, 24060

* These authors contributed equally

# Co-corresponding authors: Qiu yqiu16@umd.edu Wang B. 51022080@qq.com Wang Z. wangzhaohua@bit.edu.cn

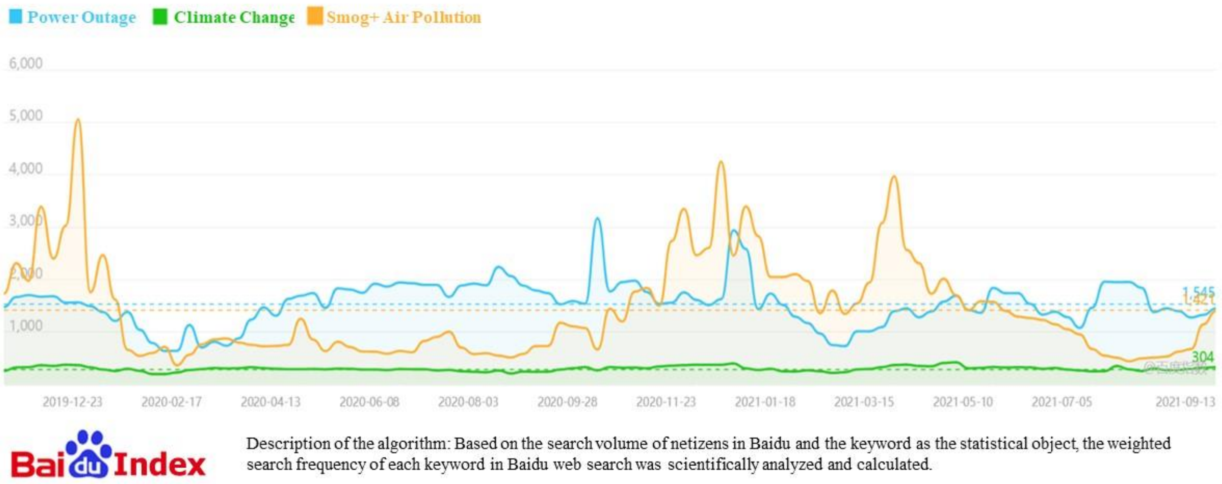

Supplementary Figure 1. Baidu keywords search index trends between three keywords: power outage, smog and air pollution, and climate change. The solid line represents the Baidu index, and the dashed line represents the mean. The blue lines refer to power outages; the green lines refer to climate change; and the orange line refers to smog and air pollution. The time range is November 2019 to September 2021. Source: Baidu Index.

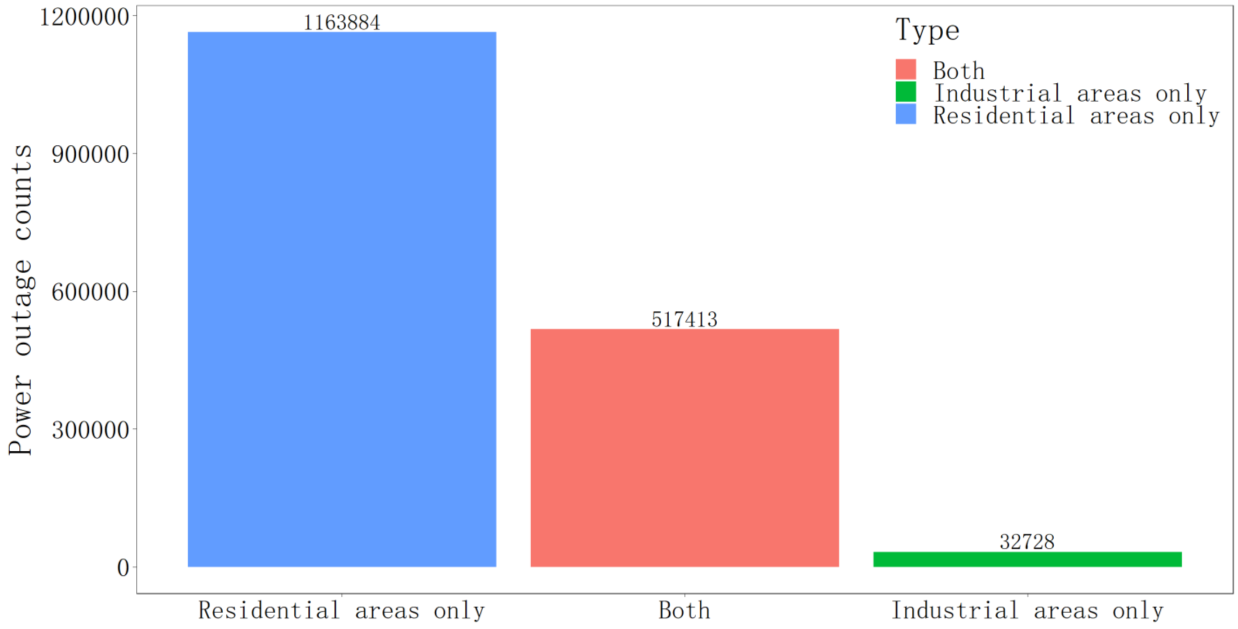

Supplementary Figure 2. The distribution of power outages in residential versus industrial areas in our dataset. The blue bars represent the number of power outages occurring exclusively in residential areas; the green bars indicate outages occurring only in industrial areas; the red bars represent the number of times outages occurred simultaneously in both locations. Source data for this figure are available on GitHub.

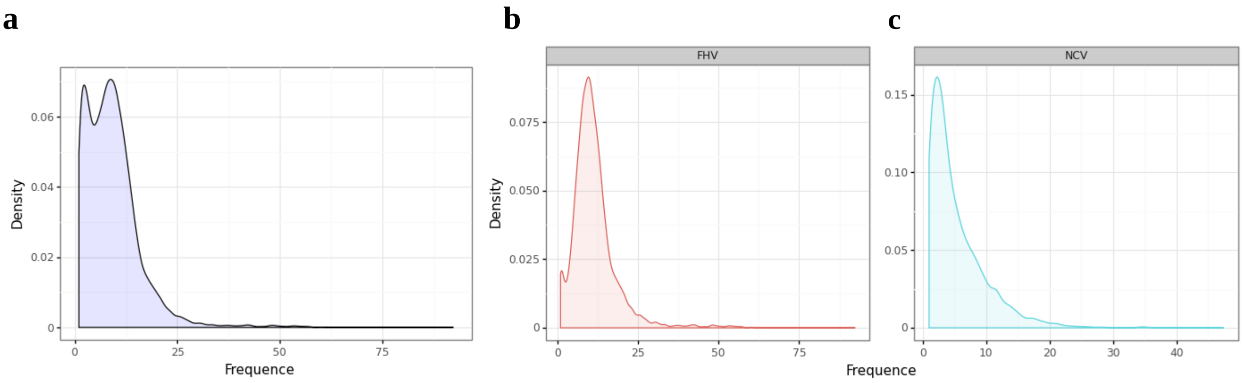

Supplementary Figure 3. **The density plot of the weekly charging frequency of EVs.** The abscissa of each subgraph is the charging frequency, the ordinate on the left is the density. The purple shade in **a** refers to all new electric vehicles, the red shade in **b** refers to for-hire vehicles (FHVs) and the blue shade in **c** refers to non-commercial vehicles (NCVs). Source data for this figure are available on GitHub.

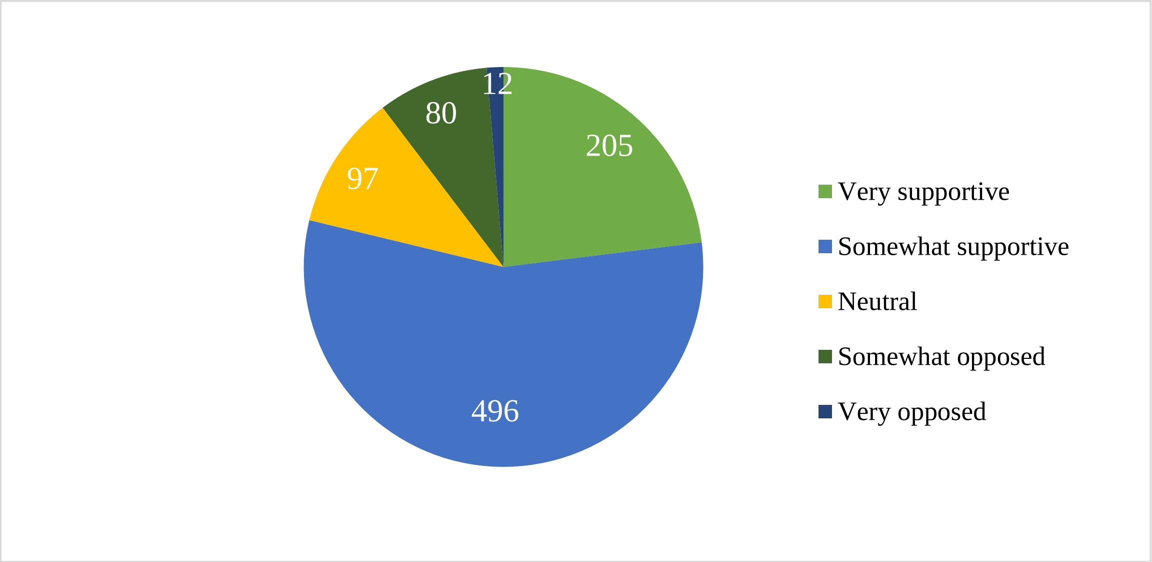

Supplementary Figure 4. **Attitudes reported by respondents in response to the statement on the impact of power outage exposure on EV purchasing intention.** The survey question is "Do you agree with the following statement: Extended power outages or increasing frequencies of power outages will reduce your willingness to purchase EVs". Source data for this figure are available on GitHub.

Supplementary Table 1. Descriptive statistics

Variables	(1) observations	(2) mean	(3) standard deviation	(4) minimum	(5) maximum
NEV	6945	404.48	1374.02	0	31489
BEV	6945	343.61	1070.60	1	19713
PHEV	6945	60.93	367.23	0	12599
NCV	6945	448.70	1356.86	0	32279
FHV	6945	61.18	247.82	0	4570
Non-EV	6945	1540.69	4370.00	1	65191
Outage times	6945	10.19	15.14	0	155.56
Outage hours	6945	103.24	252.04	0	12561.7

Note: NEV refers to new energy vehicle; BEV refers to battery electric vehicle; PHEV refers to plug-in hybrid electric vehicles; NCV refers to non-commercial vehicle; FHV refers to for-hire vehicle. Columns (1-5) show the observations, mean, standard deviation, minimum, and maximum values of the variables.

Supplementary Table 2. Regression results with EV charging station added as a control variable

	NEV sales					
	(1)	(2)	(3)	(4)	(5)	(6)
	lnNEV	lnNEV	lnBEV	lnBEV	ln PHEV	ln PHEV
L1.Outage times	-0.0099***		-0.0085***		-0.013***	
	(0.001)		(0.001)		(0.003)	
L1.Outage hours		-0.0002**		-0.0002**		-0.0003*
		(0.0001)		(0.0001)		(0.0002)
lncp	-1.35**	-1.51**	-1.38*	-1.51**	-2.46***	-2.66***
	(0.53)	(0.71)	(0.71)	(0.73)	(0.69)	(0.71)
ln(GDP)	-0.047*	-0.050*	-0.030	-0.032	-0.057	-0.060
	(0.025)	(0.026)	(0.025)	(0.025)	(0.039)	(0.040)
Constant	17.68***	19.06***	17.64***	18.83***	26.21***	28.01***
	(6.56)	(6.80)	(6.83)	(7.01)	(6.57)	(4.11)
Year*City FE	YES	YES	YES	YES	YES	YES
Month*City FE	YES	YES	YES	YES	YES	YES
Number of observations	4700	4700	4700	4700	4700	4700
Number of city	301	301	301	301	301	301
R-squared	0.90	0.90	0.91	0.90	0.74	0.73

Note: Standard errors in parentheses are clustered to city level. *P< 0.1, **P< 0.05, ***P< 0.01. R-squared denotes the goodness-of-fit of the regressions. The dependent variable in Columns (1-2) is the log of monthly sales for new energy vehicles (NEVs); in Columns (3-4), it is the log of monthly sales for battery electric vehicles (BEVs); and in Columns (5-6), it is the log of monthly sales for plug-in hybrid electric vehicles (PHEVs). Here, the total sales of NEVs equal the combined sales of both BEVs and PHEVs. L1. means one-month lag. The variable ln(GDP) is the natural log of the per capita Gross Domestic Product of each city. The variable lncp is the natural log of the provincial level EV charging stations available of a given month divided by the number of cities of the province. The EV charging station data comes from the China Electric Vehicle Charging Infrastructure Promotion Alliance (http://www.evcipa.org.cn/).

Supplementary Table 3. Regression results of the heterogeneity analysis by vehicle type using a one-month lag in power outages.

	NEV sales			
	lnNCV (non-commercial, private vehicle)		lnFHV (For-hire vehicle)	
	(1)	(2)	(3)	(4)
L1.Outage times	-0.010***		-0.002	
	(0.001)		(0.002)	
L1.Outage hours		-0.00019**		-0.00003
		(0.00009)		(0.0001)
ln(GDP)	-0.028	-0.030	-0.019	-0.019
	(0.027)	(0.028)	(0.035)	(0.035)
Constant	5.00***	4.92***	1.70**	1.68***
	(0.23)	(0.23)	(0.29)	(0.29)
Number of observations	4700	4700	4700	4700
Number of city	301	301	301	301
R-squared	0.95	0.94	0.93	0.93
Year*City FE	YES	YES	YES	YES
Month*City FE	YES	YES	YES	YES

Note: L1. means one-month lag. Standard errors in parentheses are clustered to city level. *P< 0.1, **P< 0.05, ***P< 0.01. R-squared denotes the goodness-of-fit of the regressions. The dependent variable in Columns (1-2) is the log of monthly sales for non-commercial vehicles (NCVs); in Columns (3-4), it is the log of monthly sales of for-hire vehicles (FHVs). The variable ln(GDP) is the natural log of the per capita Gross Domestic Product of each city.

Supplementary Table 4. Regression results of the heterogeneity analysis by vehicle type using a two-month lag in power outages.

	NEV sales			
	lnNCV (non-commercial vehicle)		lnFHV (For-hire vehicle)	
	(1)	(2)	(3)	(4)
L1.Outage times	-0.004***		-0.0009	
	(0.001)		(0.002)	
L1.Outage hours		-0.00006		-0.00001
		(0.00007)		(0.0001)
L2.Outage times	-0.004***		-0.004*	
	(0.001)		(0.002)	
L2.Outage hours		-0.000007		-0.00009
		(0.00005)		(0.0001)
ln(GDP)	-0.047**	-0.047**	-0.015	-0.017
	(0.020)	(0.020)	(0.038)	(0.038)
Constant	5.32***	5.24***	1.84***	1.80***
	(0.16)	(0.17)	(0.32)	(0.32)
Number of observations	4090	4090	4090	4090
Number of city	298	298	298	298
R-squared	0.92	0.92	0.83	0.82
Year*City FE	YES	YES	YES	YES
Month*City FE	YES	YES	YES	YES

Note: L1. means one-month lag; L2. means two-month lag. Standard errors in parentheses are clustered to city level. *P< 0.1, **P< 0.05, ***P< 0.01. R-squared denotes the goodness-of-fit of the regressions. The dependent variable in Columns (1-2) is the log of monthly sales for non-commercial vehicles (NCVs); in Columns (3-4), it is the log of monthly sales of for-hire vehicles (FHVs). The variable ln(GDP) is the natural log of the per capita Gross Domestic Product of each city.

Supplementary Table 5. Regression results of the heterogeneity analysis by region and GDP using a one-month lag in power outages.

	ln NEV							
	Southern provinces		Northern provinces		Low per_GDP		High per_GDP	
	(1)	(2)	(3)	(4)	(5)	(6)	(7)	(8)
L1.Outage times	-0.0096***		-0.014***		-0.014***		-0.009***	
	(0.001)		(0.003)		(0.003)		(0.001)	
L1.Outage hours		-0.00004		-0.0006***		-0.0004***		-0.00006
		(0.0001)		(0.0001)		(0.0001)		(0.0001)
ln(GDP)	-0.012	-0.011	-0.076*	-0.082**	-0.072**	-0.075**	-0.006	-0.008
	(0.035)	(0.036)	(0.041)	(0.041)	(0.035)	(0.035)	(0.041)	(0.042)
Constant	4.81***	4.66***	4.40***	4.40***	4.31***	4.28***	5.08***	4.94***
	(0.29)	(0.30)	(0.34)	(0.34)	(0.29)	(0.29)	(0.34)	(0.35)
Number of observations	2574	2574	2126	2126	2656	2656	2044	2044
Number of city	162	162	139	139	173	173	128	128
R-squared	0.96	0.96	0.96	0.96	0.96	0.96	0.96	0.96
Year*City FE	YES	YES	YES	YES	YES	YES	YES	YES
Month *City FE	YES	YES	YES	YES	YES	YES	YES	YES

Note: L1. means one-month lag. Standard errors in parentheses are clustered to city level. *P< 0.1, **P< 0.05, ***P< 0.01. R-squared denotes the goodness-of-fit of the regressions. The dependent variable in Columns (1-8) is the log of monthly sales for new energy vehicles (NEVs). The variable ln(GDP) is the natural log of the per capita Gross Domestic Product of each city.

Supplementary Table 6. Regression results of the heterogeneity analysis by region and GDP using a two-month lag in power outages.

	ln NEV							
	Southern provinces		Northern provinces		Low per_GDP		High per_GDP	
	(1)	(2)	(3)	(4)	(5)	(6)	(7)	(8)
L1.Outage times	-0.004***		-0.012***		-0.011***		-0.004***	
	(0.001)		(0.003)		(0.002)		(0.001)	
L1.Outage hours		0.00006		-0.0004***		-0.0003***		0.00002
		(0.0009)		(0.0001)		(0.0001)		(0.0001)
L2.Outage times	-0.008***		-0.013***		-0.017***		-0.006***	
	(0.001)		(0.003)		(0.003)		(0.001)	
L2.Outage hours		-0.00014		-0.0003**		-0.0003**		-0.0001
		(0.0009)		(0.0001)		(0.0001)		(0.0001)
ln(GDP)	-0.022	-0.203	-0.073**	-0.082**	-0.072**	-0.077**	-0.018	-0.017
	(0.027)	(0.029)	(0.038)	(0.037)	(0.034)	(0.034)	(0.029)	(0.031)
Constant	5.15***	4.97***	4.68***	4.63***	4.62***	4.53***	5.41***	5.24***
	(0.23)	(0.24)	(0.31)	(0.31)	(0.28)	(0.29)	(0.24)	(0.25)
Number of observations	2246	2246	1844	1844	2302	2302	1788	1788
Number of city	161	161	137	137	170	170	128	128
R-squared	0.93	0.92	0.93	0.93	0.92	0.92	0.93	0.93
Year*City FE	YES	YES	YES	YES	YES	YES	YES	YES
Month *City FE	YES	YES	YES	YES	YES	YES	YES	YES

Note: L1. means one-month lag; L2. means two-month lag. Standard errors in parentheses are clustered to city level. *P< 0.1, **P< 0.05, ***P< 0.01. R-squared denotes the goodness-of-fit of the regressions. The dependent variable in Columns (1-8) is the log of monthly sales for new energy vehicles (NEVs). The variable ln(GDP) is the natural log of the per capita Gross Domestic Product of each city. The northern versus southern provinces are divided by the Huai River. High per_GDP indicates provinces with a per capita annual GDP higher than 60,000 RMB; low per_GDP indicates provinces with a per capita annual GDP lower than 60,000 RMB.

Supplementary Table 7. Test for statistical significance of the difference between regions and GDP levels

	ln NEV			
	(1)	(2)	(3)	(4)
L1.Outage times*High GDP	0.005			
	(0.003)			
L1.Outage hours*High GDP		0.0004**		
		(0.0002)		
L1.Outage times *South			0.005	
			(0.004)	
L1.Outage hours *South				0.0005***
				(0.0001)
L1.Outage times	-0.014***		-0.014***	
	(0.003)		(0.003)	
L1.Outage hours		-0.0004***		-0.0006***
		(0.0001)		(0.0001)
ln GDP	-0.042	-0.044	-0.041	-0.043
	(0.027)	(0.027)	(0.026)	(0.027)
Constant	4.63***	4.55***	4.63***	4.54***
	(0.22)	(0.22)	(0.22)	(0.22)
Year*City FE	YES	YES	YES	YES
Month*City FE	YES	YES	YES	YES
Number of observations	4700	4700	4700	4700
Number of city	301	301	301	301
R-squared	0.96	0.98	0.96	0.96

Note: L1. means one-month lag. The northern versus southern provinces are divided by the Huai River. High GDP indicates provinces with a per capita annual GDP higher than 60,000 RMB; low GDP indicates provinces with a per capita annual GDP lower than 60,000 RMB. Standard errors in parentheses are clustered to city level. *P< 0.1, **P< 0.05, ***P< 0.01. R-squared denotes the goodness-of-fit of the regressions. The dependent variable in Columns (1-4) is the log of monthly sales for new energy vehicles (NEVs). The variable ln(GDP) is the natural log of the per capita Gross Domestic Product of each city.

Supplementary Table 8. Regression results with no lag and a three-month lag of power outages

	ln NEV			
	0-month lag	3-month lag	0-month lag	3-month lag
	(1)	(2)	(3)	(4)
Outage times	0.0013			
	(0.0012)			
Outage hours			-0.00004	
			(0.00005)	
L1.Outage times		-0.0057***		
		(0.001)		
L1.Outage hours				-0.0002**
				(0.00008)
L2.Outage times		-0.0080***		
		(0.001)		
L2.Outage hours				-0.0002***
				(0.00007)
L3.Outage times		-0.0004		
		(0.001)		
L3.Outage hours				0.000006
				(0.0001)
ln (GDP)	-0.043*	-0.049**	-0.044*	-0.049**
	(0.023)	(0.022)	(0.023)	(0.022)
lncp	-0.99**	-0.22	-0.99**	-0.30
	(0.40)	(0.51)	(0.41)	(0.52)
Constant	13.85***	7.19	13.96***	7.82
	(3.85)	(4.87)	(3.86)	(5.03)
R-squared	0.97	0.98	0.97	0.98
Year*City FE	YES	YES	YES	YES
Month*City FE	YES	YES	YES	YES
Number of observations	6304	3492	6304	3492
Number of city	304	297	304	297

Note: L1. means one-month lag; L2. means two-month lag. L3 means three-month lag. Standard errors in parentheses are clustered to city level. *P< 0.1, **P< 0.05, ***P< 0.01. R-squared denotes the goodness-of-fit of the regressions. The dependent variable in Columns (1-4) is the log of monthly sales for new energy vehicles (NEVs). The variable ln(GDP) is the natural log of the per capita Gross Domestic Product of each city. The variable lncp is the natural log of the provincial level EV charging stations available of a given month divided by the number of cities of the province.

Supplementary Table 9. Regression results of four model specifications

	Linear	Semi-log	Double-log	Exponential
	NEV	lnNEV	lnNEV	NEV
	(1)	(2)	(3)	(4)
L1.Outage times	0.88	-0.0088***		
	(0.67)	(0.001)		
ln(L1.Outage times)			-0.071***	
			(0.017)	
$e^{\text{L1.Outage times}}$				-2.49e-65***
				(3.19e-67)
ln (GDP)	9.75	-0.047**	-0.047*	9.41
	(19.26)	(0.023)	(0.025)	(19.24)
Constant	373.87**	4.85***	4.87***	386.96***
	(159.45)	(0.19)	(0.21)	(159.49)
Number of observations	4130	4130	3560	4130
Number of city	300	300	284	300
R-squared	0.97	0.98	0.96	0.97
F	1.04	25.91	10.17	3.68e+09
Year*City FE	YES	YES	YES	YES
Month*City FE	YES	YES	YES	YES

Note: L1. means one-month lag. Standard errors in parentheses; ***, **, * indicate statistical significance at 1%, 5%, and 10% levels, respectively. NEV refers to new energy vehicle. The variable ln(GDP) is the natural log of the per capita Gross Domestic Product of each city.

Supplementary Table 10. Link test results

	Linear	Semi-log	Double-log	Exponential
	(1)	(2)	(3)	(4)
yHat	-0.027	-0.82	4.06***	0.0067**
	(0.030)	(0.533)	(1.540)	(0.003)

Notes: ***, **，* indicate statistical significance at 1%, 5%, and 10% levels, respectively. Standard errors are in parentheses. yHat is the predicted value of the dependent variable (monthly sales for new energy vehicles) from each functional form.

Supplementary Table 11. Panel unit root test results

	HT (Harris and Tzavalis)	H0：Panels contain unit roots	IPS (Im, Pesaran, and Shin)	H0：Panels contain unit roots
lnBPHEV	0.76*** (0.00)	Reject	-5.71*** (0.00)	Reject
lnBEV	0.73*** (0.00)	Reject	-3.49*** (0.00)	Reject
lnPHEV	0.71*** (0.00)	Reject	-3.43*** (0.00)	Reject
Outage times	0.22*** (0.00)	Reject	-6.91*** (0.00)	Reject
Outage hours	0.07*** (0.00)	Reject	-6.26*** (0.00)	Reject
ln(GDP)	0.63*** (0.00)	Reject	-5.81*** (0.00)	Reject
lncp	0.99 (1.00)	Support	17.09 (1.00)	Support
d.lncp	-0.121*** (0.00)	Reject	-6.91*** (0.00)	Reject

Notes: P-values in parentheses; ***, **,* indicate statistical significance at 1%, 5%, and 10% levels, respectively. BPHEV refers to new energy vehicle which includes both battery electric vehicle (BEV) and plug-in hybrid electric vehicle (PHEV). The variable ln(GDP) is the natural log of the per capita Gross Domestic Product of each city. The variable lncp is the natural log of the provincial level EV charging stations available of a given month divided by the number of cities of the province. d.lncp refers to the first difference of charging stations.

Supplementary Table 12. Regression results with EV charging station (first difference) added as a control variable

	d.lnNEV<br>(1)	d.lnBEV<br>(2)	d.lnPHEV<br>(3)
L1.(d.Outage times)	-0.003***	-0.003**	-0.007***
	(0.001)	(0.001)	(0.002)
L2.(d.Outage times)	-0.008***	-0.007***	-0.02***
	(0.001)	(0.002)	(0.003)
d.ln(GDP)	-0.088***	-0.073**	-0.041
	(0.032)	(0.026)	(0.045)
d.lncp	-0.78	-0.65	-0.45
	(0.49)	(0.57)	(0.66)
Constant	0.23***	0.20***	0.34***
	(0.02)	(0.02)	(0.02)
Year*City FE	YES	YES	YES
Month*City FE	YES	YES	YES
Number of observations	2616	2616	2616
Number of city	218	218	218
R-squared	0.57	0.55	0.56

Note: L1. means one-month lag; L2. means two-month lag. Standard errors in parentheses; ***, **, * indicate statistical significance at 1%, 5%, and 10% levels, respectively. d. refers to the first-differenced number of a variable. NEV refers to new energy vehicle which includes both battery electric vehicle (BEV) and plug-in hybrid electric vehicle (PHEV). The variable ln(GDP) is the natural log of the per capita Gross Domestic Product of each city. The variable lncp is the natural log of the provincial level EV charging stations available of a given month divided by the number of cities of the province.

Supplementary Table 13. Panel cointegration test results

	Test	Statistic	P-value	H0: No cointegration
Pedroni	Modified Phillips-Perron	2.1982	0.0140	Reject
	Phillips-Perron	-28.9113	0.0000	Reject
	Augmented Dickey-Fuller	-38.0090	0.0000	Reject
Kao	Modified Dickey-Fuller	-2.0532	0.0200	Reject
	Dickey-Fuller	-14.2304	0.0000	Reject
	Augmented Dickey-Fuller	-2.0242	0.0215	Reject
	Unadjusted modified Dickey-Fuller	-47.3051	0.0000	Reject
	Unadjusted Dickey-Fuller	-37.9558	0.0000	Reject

Supplementary Table 14. Granger non-causality test for the impact of EV sales on power outages

	Statistic	P-value	Test result
lnBPHEV does not Granger-cause outaget	0.2689	0.7880	Support
lnBEV does not Granger-cause outaget	1.4045	0.1602	Support
lnPHEV does not Granger-cause outaget	1.0044	0.3152	Support
lnBPHEV does not Granger-cause outageh	-0.7011	0.4833	Support
lnBEV does not Granger-cause outageh	-0.7473	0.4549	Support
lnPHEV does not Granger-cause outageh	0.0713	0.9431	Support

Notes: BPHEV refers to new energy vehicle which includes both battery electric vehicle (BEV) and plug-in hybrid electric vehicle (PHEV). outaget refers to power outage times; outageh refers to power outage hours.

Supplementary Table 15. Granger non-causality test for the impact of power outages on EV sales

	Statistic	P-value	Test result
outaget does not Granger-cause lnBPHEV.	4.1981	0.0000	Reject
outaget does not Granger-cause lnBEV	2.6127	0.0090	Reject
outaget does not Granger-cause lnPHEV	3.6851	0.0000	Reject
outageh does not Granger-cause lnBPHEV.	4.0245	0.0001	Reject
outageh does not Granger-cause lnBEV	2.7938	0.0052	Reject
outageh does not Granger-cause lnPHEV	3.5952	0.0001	Reject

Notes: BPHEV refers to new energy vehicle which includes both battery electric vehicle (BEV) and plug-in hybrid electric vehicle (PHEV). outaget refers to power outage times; outageh refers to power outage hours.

Supplementary Table 16. Results of models adding the mandatory notice variable

	lnNEV<br>(1)	lnBEV<br>(2)	lnPHEV<br>(3)
L1.Outage times	-0.010***	-0.009***	-0.014***
	(0.001)	(0.001)	(0.003)
Ln(GDP)	-0.041	-0.023	-0.046
	(0.027)	(0.026)	(0.040)
L1.Mandatory notices	-0.48***	-0.43***	-0.58***
	(0.14)	(0.13)	(0.28)
Constant	4.63***	4.31***	2.47***
	(0.22)	(0.21)	(0.34)
Year*City FE	YES	YES	YES
Month*City FE	YES	YES	YES
Number of observations	4700	4700	4700
Number of city	301	301	301
R-squared	0.96	0.96	0.90

Notes: L1. means one-month lag. Standard errors in parentheses; ***, **, * indicate statistical significance at 1%, 5%, and 10% levels, respectively. NEV refers to new energy vehicle which includes both battery electric vehicle (BEV) and plug-in hybrid electric vehicle (PHEV). The variable ln(GDP) is the natural log of the per capita Gross Domestic Product of each city.

Supplementary Table 17. Regression results of models adding supply chain disruption and COVID restriction variables

	lnNEV			
	(1) One-month lag	(2) Two-month lag	(3) One-month lag	(4) Two-month lag
L1.Outage times	-0.0060*** (0.0011)	-0.004*** (0.001)		
L1.Outage hours			-0.00019** (0.00008)	-0.00016** (0.00008)
L2.Outage times		-0.007*** (0.001)		
L2.Outage hours				-0.00019*** (0.00006)
ln(GDP)	-0.029 (0.019)	-0.038* (0.019)	-0.029 (0.019)	-0.038* (0.020)
COVID restrictions	-0.74*** (0.052)	-0.33*** (0.051)	-0.74*** (0.052)	-0.34*** (0.050)
Disruptions in vehicle supply	-0.30*** (0.040)	-0.29*** (0.041)	-0.32*** (0.040)	-0.31*** (0.041)
Constant	4.62*** (0.16)	4.89*** (0.16)	4.59*** (0.16)	4.81*** (0.16)
Year*City FE	YES	YES	YES	YES
Month*City FE	YES	YES	YES	YES
Number of observations	4700	4090	4700	4090
Number of city	301	298	301	298
R-squared	0.97	0.98	0.97	0.98

Notes: L1. means one-month lag; L2. means two-month lag. Standard errors in parentheses; ***, **, * indicate statistical significance at 1%, 5%, and 10% levels, respectively. NEV refers to new energy vehicle. The variable ln(GDP) is the natural log of the per capita Gross Domestic Product of each city.

Supplementary Table 18. Estimation results with instrumental variable (using outage times as the explanatory variable)

	lnNEV		lnBEV		lnPHEV	
	(1)	(2)	(3)	(4)	(5)	(6)
	IV, first stage	IV, second stage	IV, first stage	IV, second stage	IV, first stage	IV, second stage
L1.(DD)	-0.33***		-0.33***		-0.33***	
	(0.033)		(0.033)		(0.033)	
L1.(outage times)		-0.13***		-0.11***		-0.16***
		(0.014)		(0.013)		(0.018)
ln(GDP)	0.18	-0.023	0.18	-0.008	0.18	-0.020
	(0.28)	(0.040)	(0.28)	(0.036)	(0.28)	(0.052)
Year*City FE	YES	YES	YES	YES	YES	YES
Month*City FE	YES	YES	YES	YES	YES	YES
Observations	4604	4604	4604	4604	4604	4604
Stock-Yogo weak ID test	16.38		16.38		16.38	
F Statistics	99.38		99.38		99.38	

Note: L1. means one-month lag. Numbers in [] are the Stock-Yogo weak ID test critical values at 10% level; the value of F statistics shows the F test of excluded instruments; numbers in () are standard errors of the coefficients. NEV refers to new energy vehicle which includes both battery electric vehicle (BEV) and plug-in hybrid electric vehicle (PHEV). DD indicates the monthly extreme temperature days variable. We use it as an instrumental variable (IV) for power outage. The variable ln(GDP) is the natural log of the per capita Gross Domestic Product of each city.

Supplementary Table 19. Estimation results with instrumental variable (using outage hours as the explanatory variable)

	lnNEV		lnBEV		lnPHEV	
	(1)	(2)	(3)	(4)	(5)	(6)
	IV, first stage	IV, second stage	IV, first stage	IV, second stage	IV, first stage	IV, second stage
L1. (DD)	-2.13^{***}		-2.13^{***}		-2.13^{***}	
	(0.45)		(0.45)		(0.45)	
L1.(outage hours)		-0.020^{***}		-0.018^{***}		-0.025^{***}
		(0.004)		(0.004)		(0.005)
ln(GDP)	1.52	-0.016	1.52	-0.002	1.52	-0.011
	(4.55)	(0.092)	(4.55)	(0.081)	(4.55)	(0.12)
Year*City FE	YES	YES	YES	YES	YES	YES
Month*City FE	YES	YES	YES	YES	YES	YES
Observations	4604	4604	4604	4604	4604	4604
Stock-Yogo weak ID test	16.38		16.38		16.38	
F statistics	22.94		22.94		22.94	

Note: L1. means one-month lag. Numbers in [] are the Stock-Yogo weak ID test critical values at 10% level; the value of F statistics shows the F test of excluded instruments; numbers in () are standard errors of the coefficients. NEV refers to new energy vehicle which includes both battery electric vehicle (BEV) and plug-in hybrid electric vehicle (PHEV). DD indicates the monthly extreme temperature days variable. We use it as an instrumental variable (IV) for power outage. The variable ln(GDP) is the natural log of the per capita Gross Domestic Product of each city.

Supplementary Table 20. Regression results with per capita EV sales and per capita power outage

	per capita sales			
	ln (per capita NEV)		ln (per capita BEV)	
	(1)	(2)	(3)	(4)
per capita outage times	0.36		0.27	
	(0.54)		(0.29)	
per capita outage hours		-0.035		-0.005
		(0.036)		(0.011)
L1. (per capita outage times)	-1.01^{**}		-0.77^{***}	
	(0.41)		(0.20)	
L1. (per capita outage hours)		-0.051		-0.013
		(0.035)		(0.015)
L2. (per capita outage times)	-1.41^{***}		-1.82^{***}	
	(0.46)		(0.39)	
L2. (per capita outage hours)		-0.001		-0.036^{**}
		(0.019)		(0.018)
ln(GDP)	-0.019	-0.020	-0.026	-0.027
	(0.027)	(0.027)	(0.022)	(0.023)
Constant	-1.08^{***}	-1.10^{***}	-1.26^{***}	-1.31^{**}
	(0.22)	(0.22)	(0.19)	(0.19)
Year*City FE	YES	YES	YES	YES
Month*City FE	YES	YES	YES	YES
Number of observations	3776	3776	3776	3776
Number of city	272	272	272	272
R-squared	0.92	0.92	0.97	0.97

Note: L1. means one-month lag; L2. means two-month lag. Standard errors in parentheses are clustered to city level. *P< 0.1, **P< 0.05, ***P< 0.01. R-squared denotes the goodness-of-fit of the regressions. NEV refers to new energy vehicle; BEV refers to battery electric vehicle. The variable ln(GDP) is the natural log of the per capita Gross Domestic Product of each city.

Supplementary Table 21. Regression results of the impact of power outages on the economy.

	ln(Industrial value added)		ln(GDP)	
outage times	-0.00004		-0.0012	
	(0.00008)		(0.0017)	
outage hours		-2.24e-06		-0.00009
		(2.93e-06)		(0.0006)
Constant	15.85^{***}	15.85^{***}	8.31^{***}	8.31^{***}
	(0.006)	(0.006)	(0.018)	(0.018)
R-squared	0.52	0.82	0.82	0.82
Time FE	YES	YES	YES	YES
City FE	YES	YES	YES	YES
Number of observations	6255	6255	6304	6304
Number of city	283	283	304	304

Note: Standard errors in parentheses are clustered to city level. *P< 0.1, **P< 0.05, ***P< 0.01. R-squared denotes the goodness-of-fit of the regressions. The variable ln(GDP) is the natural log of the per capita Gross Domestic Product of each city.

Supplementary Table 22. Between-group Difference Test

	BEV	PHEV	T-statistic (P-value）
L1.Outaget	-0.0092	-0.014	1.49 (0.14)
L1.Outageh	-0.0002	-0.0003	0.42 (0.67)

Note: L1. means one-month lag. BEV refers to battery electric vehicle; PHEV refers to plug-in hybrid electric vehicle. outaget refers to power outage times; outageh refers to power outage hours. We used T-test to whether PHEV may be more negatively impacted by power outages. The T-test was used to compare the difference between the two groups of regression coefficients. The results show that there was no significant difference between the coefficients (L1.outaget and L1.outageh) of BEV and PHEV.

Supplementary Table 23. Regression results with more socio-economical variables.

	lnNEV	lnBEV	lnPHEV
	(1)	(2)	(3)
L1. (outage times)	-0.0033***	-0.0025***	-0.0029***
	(0.0007)	(0.0007)	(0.0007)
**Socio-economical controls**	YES	YES	YES
Constant	6.86***	6.09***	15.17***
	(2.29)	(2.51)	(3.78)
Year FE	YES	YES	YES
Month FE	YES	YES	YES
City FE	YES	YES	YES
Number of observations	4842	4842	4842
Number of city	267	267	267
R-squared	0.92	0.92	0.78

Note: L1. means one-month lag. Standard errors in parentheses are clustered to city level. *P< 0.1, **P< 0.05, ***P< 0.01. R-squared denotes the goodness-of-fit of the regressions. NEV refers to new energy vehicle; BEV refers to battery electric vehicle; PHEV refers to plug-in hybrid electric vehicle. Socio-economical controls include population, per capita GDP, area of paved roads, coal gas supply, petroleum gas supply, number teachers in ordinary higher education institutions, number of public buses and the number of EV charging stations.

Supplementary Table 24. Regression results without cities significantly affected by COVID-19

	lnNEV	lnBEV	lnPHEV
	(1)	(2)	(3)
L1. (outage times)	-0.0096***	-0.0082***	-0.012***
	(0.0015)	(0.0014)	(0.0030)
lnGDP	-0.031	-0.014	-0.039
	(0.029)	(0.028)	(0.040)
Constant	4.36***	4.05***	2.24***
	(0.24)	(0.23)	(0.33)
Year*City FE	YES	YES	YES
Month*City FE	YES	YES	YES
Number of observations	3754	3754	3754
Number of city	241	241	241
R-squared	0.89	0.89	0.75

Note: L1. means one-month lag. NEV refers to new energy vehicle; BEV refers to battery electric vehicle; PHEV refers to plug-in hybrid electric vehicle. Standard errors in parentheses are clustered to city level. *P< 0.1, **P< 0.05, ***P< 0.01. R-squared denotes the goodness-of-fit of the regressions. We excluded 61 cities that were listed as medium or high-risk areas during the pandemic.

Supplementary Table 25. The impact of power outage on EV purchase intention using survey data

	EV purchase intention	EV purchase intention
	(1)	(2)
power outage level	-0.052**	
	(0.022)	
power outage times		-0.054***
		(0.011)
income level	0.083***	0.22***
	(0.016)	(0.019)
education level	-0.048	-0.036
	(0.048)	(0.060)
charging infrastructure reliability	0.089***	0.55***
	(0.033)	(0.029)
home charging concern	-0.16***	-0.049***
	(0.017)	(0.025)
public perception	0.018	-0.0072
	(0.018)	(0.024)
road infrastructure access	0.055*	0.033
	(0.029)	(0.034)
government incentive	0.056**	0.11***
	(0.026)	(0.036)
environmental awareness	-0.018	0.053**
	(0.028)	(0.023)
urban congestion	-0.33***	0.0072
	(0.020)	(0.034)
access to public transport	0.018	0.026
	(0.024)	(0.029)
perception of low carbon technologies	0.0010	0.0048
	(0.027)	(0.026)
range anxiety	-0.16***	-0.15***
	(0.023)	(0.036)
City FE	YES	YES
Observations	976	856
R-squared	0.80	0.72

Note: Standard errors in parentheses are clustered to individual level. *P< 0.1, **P< 0.05, ***P< 0.01. R-squared denotes the goodness-of-fit of the regressions. EV refers to the new energy vehicle.

Supplementary Note 1. Explanations of the fixed effects in the main model

Based on the previous studies[1,2], we include city by month fixed effect and city by year fixed effect in our regression model, denoted by λ_{im} and θ_{iy}, respectively. City by month fixed effect flexibly controls for month-specific shocks in each city, such as climatic conditions. City by year fixed effect controls for year specific shocks in each city, such as city scale, subsidies, income, and macroeconomic trends. To further clarify, for each city and each year, there will be a separate dummy variable included in the regression model (except for the base case) and that dummy variable will control for these spatial differences that may also change over time. Similarly, for each city and each month of the year, there will be a separate dummy variable included in the regression model and that dummy variable will control for spatial differences by season.

Supplementary References

1. Tanaka, T., & Okamoto, S. Increase in suicide following an initial decline during the COVID-19 pandemic in Japan. Nat. Hum. Behav. 5(2), 229-238 (2021).

2. Burke, M., González, F., Baylis, P., Heft-Neal, S., Baysan, C., Basu, S., & Hsiang, S. Higher temperatures increase suicide rates in the United States and Mexico. Nat. Clim. Change, 8(8), 723-729 (2018).
